# Clinical Outcomes of Aerosolized Versus Intravenous Colistin in Ventilator-Associated Pneumonia Caused by Multidrug-Resistant Gram-Negative Bacteria

**DOI:** 10.14740/jocmr6415

**Published:** 2026-01-16

**Authors:** Do Van Loi, Luu Thuy Hien, Tran Thi Tuoi, Nguyen Phuc Thanh, Tran Vuong The Vinh, Luu Quang Thuy, Le Thi Nguyet

**Affiliations:** a Department of Anesthesia, School of Medicine and Pharmacy, Phenikaa University, Hanoi, Vietnam; b Anesthesiology Center, Phenikaa University Hospital, Hanoi, Vietnam; c Department of Surgery, Hai Phong University of Medicine and Pharmacy, Hai Phong, Vietnam; d Anesthesiology Center, Viet Duc Hospital, Hanoi, Vietnam

**Keywords:** Aerosol therapy, Critical care, Drug delivery system, Nephrotoxicity, Ventilator-associated pneumonia

## Abstract

**Background:**

Ventilator-associated pneumonia (VAP) caused by multidrug-resistant (MDR) Gram-negative bacteria has presented significant treatment challenges in critical care. While intravenous colistin is commonly used, its nephrotoxicity and limited lung penetration raise concerns. This study aimed to compare the clinical efficacy and safety of aerosolized versus intravenous colistin in patients with VAP.

**Methods:**

The study included 60 adult patients diagnosed with VAP caused by colistin-sensitive MDR Gram-negative bacteria. Treatment decisions (aerosolized or intravenous colistin) were made by attending physicians based on clinical judgment (n = 30 per each group). The primary outcome was clinical success; secondary outcomes included time to defervescence, Clinical Pulmonary Infection Score changes, and adverse events.

**Results:**

Clinical success was achieved in 80.0% of patients in the aerosolized group compared with 70.0% in the intravenous group (P = 0.38). The time to defervescence was significantly shorter in the aerosolized group (3.0 ± 1.2 days) than in the intravenous group (5.0 ± 1.7 days; P = 0.002). Nephrotoxicity occurred in 13.3% of patients receiving aerosolized colistin and in 23.3% of those receiving intravenous colistin (odds ratio (OR) 0.51; 95% confidence interval (95% CI) 0.13–2.03; P = 0.19). Microbiological clearance was observed in 66.7% of the aerosolized group and 56.7% of the intravenous group (P = 0.44). Intensive care unit mortality was 16.7% in the aerosolized group and 23.3% in the intravenous group (P = 0.52).

**Conclusion:**

Aerosolized colistin was feasible and generally well tolerated; however, these findings should be interpreted as descriptive and hypothesis-generating, and further studies are needed to confirm their clinical relevance.

## Introduction

Ventilator-associated pneumonia (VAP) remains a major cause of morbidity and mortality in intensive care units (ICUs) worldwide. Its burden is especially pronounced in low- and middle-income countries, where ICU infrastructure and access to newer antibiotics are often limited. Among these, Southeast Asia has emerged as a hotspot for antimicrobial resistance, with particularly high rates of multidrug-resistant (MDR) Gram-negative pathogens, including *Acinetobacter baumannii*, *Pseudomonas aeruginosa*, and *Klebsiella pneumoniae* [1–3].

In this region, carbapenem resistance has reached alarming levels, leaving colistin, a polymyxin antibiotic, as one of the few viable treatment options for VAP. However, intravenous colistin is associated with suboptimal lung penetration and a high risk of nephrotoxicity, complications that are particularly concerning in resource-limited critical care settings with constrained renal replacement therapy capacity. These factors underscore the need for optimized, safer delivery methods of colistin that maximize pulmonary efficacy while minimizing systemic toxicity [4–7].

To address these concerns, aerosolized colistin has been proposed as an alternative administration route that allows high local drug concentrations at the site of infection while minimizing systemic exposure and associated toxicities. Preliminary studies have shown favorable pharmacokinetics and potential safety advantages of inhaled colistin [8–10]. Despite this, comparative clinical data on aerosolized versus intravenous colistin remain limited, particularly in real-world settings in Southeast Asia, where the clinical need is most urgent [11].

This study aimed to evaluate the clinical efficacy and renal safety of aerosolized versus intravenous colistin in Vietnamese ICU patients with VAP caused by MDR Gram-negative bacteria. We hypothesized that aerosolized colistin could provide comparable or superior therapeutic outcomes while reducing nephrotoxicity. By generating region-specific data, this study seeks to fill an important evidence gap and support more context-appropriate treatment strategies for MDR-VAP in resource-constrained settings like Vietnam and the broader Southeast Asian region. The findings of this study would be expected to provide valuable evidence to inform clinical decision-making and contribute to optimizing therapeutic strategies for VAP.

## Materials and Methods

### Study design and subjects

This observational cohort study was conducted in the ICU II of Viet Duc Hospital, Hanoi, Vietnam, from January 2022 to June 2022. The study adhered to ethical guidelines and received approval from the Hanoi Medicine University Institutional Review Board (5563/QD-DHYHN).

### Inclusion criteria

Patients were eligible for enrollment if they met all of the following criteria: 1) adults aged 18 years or older; 2) diagnosed with VAP based on clinical, radiological, and microbiological criteria, including a Clinical Pulmonary Infection Score (CPIS) greater than 6 (Supplementary Material 1, jocmr.elmerjournals.com); 3) required invasive mechanical ventilation for at least 48 h prior to the diagnosis of VAP; 4) microbiological confirmation of infection by MDR Gram-negative bacteria, as evidenced by positive cultures from lower respiratory tract specimens; 5) bacterial isolates sensitive to colistin, based on in vitro susceptibility testing; 6) expected to remain on mechanical ventilation for a minimum of 72 h after initiation of colistin therapy; and 7) provision of written informed consent by the patient or legally authorized representative.

### Exclusion criteria

Patients were excluded from the study if they met any of the following criteria: 1) pregnant or breastfeeding women; 2) patients with confirmed or suspected acute renal failure at the time of enrollment (defined as serum creatinine > 2 mg/dL or those requiring renal replacement therapy); 3) known hypersensitivity or contraindication to colistin or any component of the formulation; 4) use of colistin (intravenous or aerosolized) within 14 days prior to enrollment; 5) receipt of colistin therapy for less than 72 h after enrollment; 6) concomitant enrollment in another interventional trial; 7) severe immunosuppression, defined as absolute neutrophil count < 500 cells/mm^3^, use of high-dose corticosteroids (equivalent to ≥ 1 mg/kg/day of prednisone for ≥ 2 weeks), or ongoing chemotherapy; and 8) patients with end-of-life care status or do-not-resuscitate orders at the time of enrollment.

### Sample size calculation

The sample size for this study was determined based on the expected difference in clinical response rates between the two treatment groups, using data from a previous study by Abdellatif et al [12]. In that study, the clinical response rate in the aerosolized colistin group was reported as 84%, whereas the intravenous colistin group demonstrated a response rate of 58%. These proportions (p_1_ = 0.84 and p_2_ = 0.58) were used for sample size estimation.

The following formula for comparing two proportions was applied:

$$n=\frac{{\left(Z_{\alpha /2}+Z_{\beta }\right)}^{2}\times \left[p_{1}\left(1\;-\;p_{1}\right)+p_{2}\left(1-p_{2}\right)\right]}{{\left(p_{1}-p_{2}\right)}^{2}}$$

where Z_α/2_ = 1.96, corresponding to a two-sided significance level (α) of 0.05. Z_β_ = 1.28, corresponding to a desired statistical power (1 − β) of 90%. p_1_ = 0.84 (expected proportion in the aerosolized group). p_2_ = 0.58 (expected proportion in the intravenous group).

The pooled estimated proportion p was calculated as:

$$p=\frac{p_{1}+\;p_{2}}{2}=0.71$$

The calculated minimum sample size per group was approximately 29 patients. To ensure adequate power and account for potential dropouts, the final sample size was rounded up to 30 patients per group. Thus, a total of 60 patients were enrolled in the aerosolized and intravenous groups.

### Group allocation and treatment protocol

This study was conducted as an observational cohort study without randomization. Eligible patients who received either aerosolized or intravenous colistin as part of routine ICU care were consecutively screened and enrolled. Treatment allocation was entirely determined by the attending ICU physicians based on clinical judgment, patient condition, and institutional protocols, without any involvement or influence from the research team.

To ensure balanced comparison between groups, enrollment was prospectively capped at 30 patients per treatment arm, in accordance with the predefined sample size calculation. Once the required number of patients was reached in each group, further eligible patients were not included. No matching, crossover, or reassignment between groups was performed at any stage.

In routine clinical practice, aerosolized colistin was more commonly prescribed for patients considered to be at higher risk of renal impairment or those expected to benefit from targeted pulmonary drug delivery, whereas intravenous colistin was selected in other clinical scenarios at the discretion of the treating physician. Patients were subsequently classified into study groups solely based on the route of colistin administration received: 1) Aerosolized group (n = 30): Patients received 2 million international units (MIU) of colistin dissolved in 10 mL of sterile saline, administered via a vibrating mesh nebulizer every 6 h for 30 min. The nebulizer was placed approximately 10 cm from the Y-piece of the ventilator circuit with humidification turned off during the procedure. 2) Intravenous group (n = 30): Patients received a loading dose of 9 MIU colistin, followed by a maintenance dose of 2 MIU diluted in 50 mL of saline and infused intravenously over 60 min every 6 h.

### Diagnosis and treatment protocol

After admission to ICU, patients received general management including mechanical ventilation and intensive supportive care for various underlying conditions such as traumatic brain injury, subarachnoid hemorrhage, and septic shock. Comprehensive daily care was provided, which included treatment of the underlying disease, empirical antibiotic therapy, close monitoring of clinical status, and routine laboratory investigations.

Patients who developed signs of pneumonia after more than 48 h of mechanical ventilation were evaluated for inclusion in the study. The diagnostic criteria for VAP included the following: 1) fever: body temperature above 38 °C; 2) increased and purulent bronchial secretions (observed either through spontaneous coughing or suctioning, with yellow, thick, and copious sputum); 3) elevated or decreased peripheral white blood cell count (white blood cell (WBC) > 10 × 10^9^/L or < 5 × 10^9^/L); and 4) new onset of infiltrates or consolidation on chest X-ray, either localized or bilateral, suggestive of pneumonia or bronchopneumonia.

For microbiological confirmation, bronchial secretions were collected using a “blind” distal sampling technique through a double-lumen closed suction catheter inserted via the endotracheal or tracheostomy tube. Samples were cultured, and antimicrobial susceptibility testing was performed.

Patients with microbiological evidence of Gram-negative bacteria sensitive to colistin were eligible for inclusion. Antibiotic regimens were administered according to the susceptibility profiles: 1) For isolates sensitive to colistin and at least one other antibiotic, colistin was administered in combination with the susceptible agent. 2) For multidrug-resistant Gram-negative bacteria sensitive only to colistin, colistin was combined with a carbapenem.

From the initiation of colistin therapy (day 0), patients were monitored until clinical resolution, discharge, voluntary withdrawal, or death. Data collection included: 1) baseline patient characteristics: severity scores, sequential organ failure assessment (SOFA), CPIS, Acute Physiology and Chronic Health Evaluation II (APACHE II), chest X-ray findings, microbiological data, comorbidities, and other relevant clinical information; 2) clinical monitoring: vital signs (heart rate, temperature, blood pressure), use of vasopressors, FiO_2_, fluid balance (input/output), quantity and characteristics of sputum, and daily assessment of SOFA and CPIS scores; 3) laboratory parameters: WBC count, C-reactive protein, blood urea nitrogen, creatinine, bilirubin, arterial blood gas analysis, and chest radiography; and 4) microbiological follow-up: repeat cultures of bronchial secretions were performed if clinical deterioration or lack of improvement was observed.

Baseline renal function, including serum creatinine levels and absence of acute renal failure at enrollment, was documented for all patients and did not differ significantly between groups at the time of colistin initiation.

### Adjunctive antibiotic therapy

In addition to colistin, adjunctive antibiotics were prescribed according to microbiological susceptibility results and institutional treatment protocols. The choice of accompanying antibiotics, including carbapenems, tigecycline, aminoglycosides, vancomycin, or quinolones, was made at the discretion of the treating physician based on pathogen profile and clinical severity.

For the purpose of this study, adjunctive antibiotics were recorded and analyzed by antibiotic class only, rather than by individual agent, dose, or duration. This approach was adopted because dosing regimens and treatment duration were individualized according to patient condition, renal function, and antimicrobial susceptibility, and were not intended as comparative exposure variables between groups.

### Outcomes

#### Primary outcome

The primary endpoint of the study was clinical response, assessed at the end of colistin therapy. Clinical response was categorized into four groups: 1) Clinical cure: complete resolution of VAP signs and symptoms, including: body temperature ≤ 38°C; decrease or absence of purulent bronchial secretions; radiological improvement on chest X-ray (partial or complete resolution of infiltrates); successful weaning from mechanical ventilation without recurrence. 2) Clinical improvement: partial resolution of symptoms without full recovery, allowing continued therapy or de-escalation. 3) Clinical failure: persistence or worsening of clinical signs requiring a change in antibiotic therapy or resulting in death. 4) Death: all-cause mortality during ICU stay, regardless of direct relation to pneumonia.

For analysis, clinical success was defined as achieving either cure or improvement, while failure and death were classified as unsuccessful outcomes.

#### Secondary outcomes

Secondary endpoints included the following clinical and paraclinical parameters: 1) Time to defervescence: number of days from colistin initiation to normalization of temperature (≤ 38 °C) maintained for ≥ 48 h; 2) Changes in CPIS scores: CPIS evaluated at day 0 (baseline), day 7, and day 14 of therapy to assess progression of infection; 3) Nephrotoxicity: defined and graded according to the RIFLE (Risk, Injury, Failure, Loss, End-stage kidney disease) classification, based on changes in serum creatinine or urine output during treatment; 4) All-cause ICU mortality: death during ICU admission, irrespective of VAP resolution status; 5) Duration of mechanical ventilation: total number of ventilator days during ICU stay; 6) Length of ICU stay: total number of days from ICU admission to discharge or death; and 7) Use of vasopressors: requirement and duration of vasopressor support during the course of treatment.

### Safety and adverse events

In addition to renal outcomes, all non-renal adverse effects were monitored and documented, including: 1) neurological symptoms (e.g., paresthesia, confusion); 2) hypersensitivity reactions; and 3) bronchospasm (in the aerosolized group).

All adverse events were assessed for severity and potential association with colistin therapy.

### Assessment of temperature and clinical response

Body temperature was measured using standardized bedside ICU monitoring systems routinely employed at the study center. Temperature recordings were obtained continuously and documented at regular intervals according to institutional ICU protocols, ensuring consistency across all enrolled patients.

Clinical response was assessed using predefined criteria and applied uniformly to both treatment groups. Clinical cure was defined as complete resolution of VAP signs and symptoms, including sustained normalization of body temperature (≤ 38 °C for at least 48 h), reduction or absence of purulent respiratory secretions, radiographic improvement on chest imaging, and successful weaning from mechanical ventilation without recurrence of infection. Clinical improvement was defined as partial resolution of these features without full recovery. All assessments were performed by the treating ICU team following standardized institutional criteria.

### Data statistics

All statistical analyses were performed using SPSS version 20.0 (IBM Corp., Armonk, NY, USA). Continuous variables were expressed as mean ± standard deviation (SD) and compared between groups using the independent samples *t*-test for normally distributed data (assessed by Shapiro–Wilk test) or the Mann–Whitney U test for non-parametric data. Categorical variables were presented as frequencies and percentages, and compared using the Chi-square test or Fisher’s exact test. For proportions, 95% confidence intervals (CIs) were calculated using the Clopper–Pearson exact method, which is suitable for small sample sizes. Odds ratios (ORs) were calculated using univariate logistic regression to explore the association between the route of colistin administration and binary outcomes, with the intravenous colistin group serving as the reference. Given the limited sample size and number of outcome events, multivariable regression analysis was not performed, as adjustment for multiple covariates (e.g., age, sex, body mass index (BMI), and comorbidities) could result in model overfitting and unstable estimates. Therefore, all ORs presented in this study should be interpreted as unadjusted and exploratory, rather than causal estimates. All statistical tests were two-tailed, and a P-value < 0.05 was considered statistically significant.

## Results

### Patient characteristics

There was no significant difference between the mean age of the aerosolized and intravenous groups (50.2 ± 20.9 vs. 53.8 ± 19.5 years, respectively; P = 0.529). Males accounted for 83.3% of the total cohort (80% in the aerosolized group and 86.7% in the intravenous group; P = 0.488). The mean height, weight, and BMI were comparable between groups (P > 0.05), indicating well-matched groups at baseline.

The primary indications for mechanical ventilation included traumatic brain injury (TBI), septic shock, polytrauma, and other conditions. TBI was the most common cause, accounting for approximately 60% of cases. There were no significant differences between groups regarding the distribution of underlying diseases (P = 0.61).

Common comorbid conditions included hypertension, diabetes mellitus, and gout. Hypertension was the most frequently observed comorbidity, present in approximately 40% of patients. No significant intergroup differences were found in the prevalence of comorbidities (P > 0.05).

The severity of illness at the initiation of colistin therapy was assessed using SOFA, APACHE II, and CPIS scores. The median SOFA scores were 5 (interquartile range (IQR): 3–8) in the aerosolized group and 5 (IQR: 2–7) in the intravenous group (P = 0.941). The mean APACHE II scores were 16.2 ± 5.2 and 15.8 ± 6.3, respectively (P = 0.419). The median CPIS scores were also similar (P = 0.427). These results indicated comparable severity between the two groups at baseline.

Microbiological examination identified *Acinetobacter baumannii*, *Klebsiella pneumoniae*, and *Pseudomonas aeruginosa* as the most common pathogens. All isolates were sensitive to colistin. There were no significant differences in the distribution of bacterial species between the two groups (P > 0.05), suggesting similar microbiological profiles across study arms.

The distribution of adjunctive antibiotics combined with colistin was similar between the aerosolized and intravenous groups. Carbapenems were the most commonly co-administered agents, used in approximately two-thirds of patients in both groups (63.3% vs. 66.7%, P > 0.05). The use of tigecycline, aminoglycosides, and vancomycin was infrequent and balanced between groups (all P > 0.05). Notably, vancomycin was used in a slightly higher proportion of patients in the aerosolized group (13.3% vs. 6.7%, P > 0.05), possibly reflecting clinician concerns about Gram-positive co-infections, although no such pathogens were detailed in the microbiological data.

Importantly, aminoglycosides, known for their nephrotoxic potential, were administered to 13.3% and 16.7% of patients in the inhalation and intravenous groups, respectively (P > 0.05) (Table 1).

**Table 1:** Patient Characteristics

	Aerosolized group (n = 30)	Intravenous group (n = 30)	P-value
Demographic characteristics			
Age (years), mean ± SD	50.2 ± 20.9	53.8 ± 19.5	0.529
Male, n (%)	24 (80%)	26 (86.7%)	0.488
Height (cm), mean ± SD	166.3 ± 7.8	167.3 ± 7.4	0.589
Weight (kg), mean ± SD	61.4 ± 11.2	62.4 ± 10.6	0.681
Body mass index (kg/m^2^), mean ± SD	22.2 ± 3.4	22.3 ± 3.1	0.892
Comorbidities			
Traumatic brain injury, No. (%)	18 (60%)	18 (60%)	0.610
Hypertension, No. (%)	12 (40%)	12 (40%)	0.890
Diabetes mellitus, No. (%)	4 (13.3%)	3 (10%)	0.689
Gout, No. (%)	3 (10%)	4 (13.3%)	0.673
Severity			
SOFA score, median (IQR)	5 (3–8)	5 (2–7)	0.941
APACHE II score, mean ± SD	16.2 ± 5.2	15.8 ± 6.3	0.419
CPIS score, median (IQR)	6 (4–7)	6 (4–7)	0.427
Microbiological characteristics			
*A. baumannii* isolated, No. (%)	20 (66.7%)	21 (70%)	0.781
*K. pneumoniae* isolated, No. (%)	5 (16.7%)	4 (13.3%)	0.709
*P. aeruginosa* isolated, No. (%)	5 (16.7%)	5 (16.7%)	1.000
Antibiotics combined			
Carbapenem	19 (63.3%)	20 (66.7%)	1.000
Tigecycline	4 (13.3%)	5 (16.7%)	1.000
Vancomycin	4 (13.3%)	2 (6.7%)	0.671
Aminoglycoside	4 (13.3%)	5 (16.7%)	1.000
Quinolone	1 (3.3%)	0 (0%)	1.000

Adjunctive antibiotics are presented as antibiotic classes used concomitantly with colistin. Individual antibiotic agents, dosing regimens, and duration of therapy were individualized according to antimicrobial susceptibility testing and clinical judgment and were therefore not compared between groups. IQR: interquartile range; SD: standard deviation.

### Clinical outcomes

Overall clinical success was achieved in 24 patients (80.0%) in the aerosolized colistin group and in 21 patients (70.0%) in the intravenous colistin group, with no statistically significant difference between groups (P = 0.552). The mean time to defervescence was shorter in the aerosolized group (3.0 ± 1.2 days) compared with the intravenous group (5.0 ± 1.7 days; P = 0.002). CPIS scores were comparable between groups at baseline (day 0). At day 7, a greater reduction in CPIS score was observed in the aerosolized group (P = 0.012); however, by day 14, CPIS values were similar between groups, with no statistically significant difference (P = 0.337). Nephrotoxicity occurred in four patients (13.3%) receiving aerosolized colistin and in seven patients (23.3%) receiving intravenous colistin (P = 0.506; unadjusted OR 0.51, 95% CI 0.13–2.03). ICU mortality was observed in five patients (16.7%) in the aerosolized group and in seven patients (23.3%) in the intravenous group (P = 0.748; unadjusted OR 0.66, 95% CI 0.18–2.42). Microbiological clearance was documented in 20 patients (66.7%) in the aerosolized group and in 17 patients (56.7%) in the intravenous group (P = 0.596; unadjusted OR 1.75, 95% CI 0.63–4.88). No statistically significant differences were observed between groups in the duration of mechanical ventilation (P = 0.572) or length of ICU stay (P = 0.150) (Table 2).

**Table 2:** Clinical Outcomes

Outcome	Aerosolized group	Intravenous group	P-value	OR (95% CI)
Time to defervescence (days)	3.0 ± 1.2	5.0 ± 1.7	0.002*	-
Clinical success	24 (80%)	21 (70%)	0.55	1.71 (0.53–5.52)
Nephrotoxicity	4 (13.3%)	7 (23.3%)	0.50	0.51 (0.13–2.03)
Mortality	5 (16.7%)	7 (23.3%)	0.75	0.66 (0.18–2.42)
Microbiological clearance	20 (66.7%)	17 (56.7%)	0.60	1.75 (0.63–4.88)
CPIS day 0	8.5 ± 1.5	8.0 ± 1.2	0.16	-
CPIS day 7	5.0 ± 1.5	6.0 ± 1.5	0.01*	-
CPIS day 14	3.5 ± 2.0	4.0 ± 2.0	0.34	-
Duration of mechanical ventilation (days)	22.4 ± 7.6	23.5 ± 7.4	0.57	-
ICU length of stay (days)	18.7 ± 8.3	21.9 ± 8.7	0.15	-

Data were presented as No. (%) or mean ± standard deviation (SD). *P < 0.05.

Clinical response increased over time in both treatment groups. At day 3, clinical response was observed in 20.0% of patients in the aerosolized group and 16.7% in the intravenous group (P = 0.877). By day 7, response rates increased to 67.3% and 50.0%, respectively (P = 0.240), and further to 76.7% versus 66.7% by day 10 (P = 0.162). The overall clinical response rate was 76.7% in the aerosolized group and 70.0% in the intravenous group, with no statistically significant difference (P = 0.081). Subgroup analyses according to microbiological pathogens showed no statistically significant differences between groups. Among patients with *Acinetobacter baumannii* infection, clinical success was observed in 68.4% of the aerosolized group and 57.1% of the intravenous group (P = 0.215; unadjusted OR 2.33, 95% CI 0.72–7.58). In cases involving *Klebsiella pneumoniae*, clinical success was achieved in all patients in both groups. For *Pseudomonas aeruginosa*, response rates were 50.0% in the aerosolized group and 42.9% in the intravenous group (P = 0.814; unadjusted OR 5.33, 95% CI 0.65–43.5) (Table 3).

**Table 3:** Clinical Response Efficacy by Time Point and Microbiological Characteristics

Clinical response	Aerosolized group	Intravenous group	P-value	OR (95% CI)
By time point				
Day 3	20.0%	16.7%	0.877	-
Day 7	67.3%	50.0%	0.240	-
Day 10	76.7%	66.7%	0.162	-
Overall	76.7%	70.0%	0.081	-
By microbiological characteristics				
*A. baumannii*	13/19 (68.4%)	12/21 (57.1%)	0.215	2.33 (0.72–7.58)
*K. pneumoniae*	3/3 (100%)	2/2 (100%)	1.000	2.25 (0.36–14.2)
*P. aeruginosa*	4/8 (50.0%)	3/7 (42.9%)	0.814	5.33 (0.65–43.5)

95% CI: 95% confidence interval; OR: odds ratio.

### Adverse effects

Renal injury was observed in 13.3% of patients (n = 4) in the aerosolized group and 23.3% in the intravenous group (n = 7, P = 0.186). Renal injury occurred in four patients (13.3%) in the aerosolized group and seven patients (23.3%) in the intravenous group. The unadjusted OR was 0.51 (95% CI 0.13–2.03), indicating a numerically lower incidence in the aerosolized group, although the CI was wide and the difference did not reach statistical significance. According to the RIFLE classification, the majority of renal events fell under the risk (R) category, 75.0% (n = 3) in the aerosolized group vs. 57.1% (n = 4) in the intravenous group (P = 0.324). The remaining events were categorized as injury (I) and failure (F), with no cases of end-stage renal disease or need for renal replacement therapy reported.

No neurological toxicity, allergic reactions, or bronchospasm were observed in either group during the study period. The absence of bronchospasm in the aerosolized group may reflect the careful administration protocol using a vibrating mesh nebulizer and pre-treatment bronchodilator use when indicated.

## Discussion

The study showed that aerosolized colistin was feasible to administer and generally well tolerated in critically ill patients with VAP caused by MDR Gram-negative bacteria, while overall clinical outcomes were comparable between treatment groups. These findings support the feasibility of aerosolized colistin in routine ICU practice but should not be interpreted as evidence of therapeutic superiority or substitution for intravenous therapy.

Patients receiving aerosolized colistin experienced a shorter time to defervescence and greater early reduction in CPIS scores. However, these findings reflect differences in the timing of selected clinical parameters rather than overall treatment effectiveness. By the end of therapy, no statistically significant differences were observed between groups in overall clinical success, microbiological clearance, duration of mechanical ventilation, length of ICU stay, or mortality [12, 13]. Accordingly, early changes in clinical parameters should be interpreted as descriptive observations rather than indicators of improved long-term outcomes.

Comparisons with prior studies and meta-analyses should be interpreted cautiously. Although several observational studies have reported similar patterns of clinical and microbiological outcomes with aerosolized colistin, heterogeneity in study design, patient populations, dosing regimens, and outcome definitions limits direct comparison [14, 15]. Importantly, evidence from the present study does not support extrapolation of non-significant findings to conclusions regarding clinical benefit or superiority, particularly in the absence of causal adjustment.

From a safety perspective, nephrotoxicity occurred numerically less frequently in the aerosolized group; however, this difference was not statistically significant and was accompanied by wide CIs. These findings should therefore be regarded as descriptive and exploratory rather than indicative of a renal protective effect. Adjunctive antibiotic exposure, including potentially nephrotoxic agents, was comparable between groups, which may reduce, but does not eliminate, the potential for confounding in renal safety assessment. No cases of neurotoxicity, hypersensitivity reactions, or bronchospasm were observed in either group, supporting the overall tolerability of both treatment approaches in this cohort [12, 16, 17].

No statistically significant differences were observed in ICU length of stay or duration of mechanical ventilation, indicating comparable clinical trajectories during the ICU course. Although earlier symptom resolution was observed in the aerosolized group, this observation was not associated with measurable differences in downstream clinical outcomes and cannot be used to infer effects on patient comfort, ventilator weaning, or secondary infection risk, which were not systematically assessed.

It is also important to emphasize that the aerosolized and intravenous colistin regimens used in this study were clinically appropriate but pharmacologically non-equivalent. The intravenous regimen included a loading dose intended to rapidly achieve systemic exposure, whereas aerosolized colistin was administered without a loading dose and primarily targeted pulmonary drug delivery. In the absence of pharmacokinetic data, including plasma or epithelial lining fluid concentrations, comparable clinical outcomes between groups should not be interpreted as evidence of equivalent drug exposure or pharmacological effect. Known differences in pharmacokinetics between inhaled and intravenous colistin may contribute to observed clinical patterns but cannot be quantified within the scope of this study [17–19].

Several limitations must be considered. The observational and non-randomized design introduces confounding by indication, as treatment allocation was based on clinician judgment. Although baseline demographic characteristics, renal function, and illness severity scores were comparable between groups at treatment initiation, unmeasured factors related to renal risk, pulmonary severity, or clinical trajectory may have influenced both treatment selection and outcomes. The limited sample size reduced statistical power and precluded multivariable adjustment, and all comparative analyses should therefore be interpreted as unadjusted and hypothesis-generating. In addition, heterogeneity in adjunctive antibiotic regimens and incomplete microbiological follow-up may have influenced outcome assessment. Post-discharge outcomes, including 30-day mortality, were not consistently available, and generalizability may be limited by the single-center design. Further adequately powered, randomized studies incorporating pharmacokinetic assessment are required to better define the role of aerosolized colistin in the management of VAP [17].

### Conclusion

Aerosolized colistin was feasible and generally well tolerated in this observational cohort of patients with VAP caused by MDR Gram-negative bacteria. However, given the observational design, limited sample size, and lack of causal adjustment, these findings should be interpreted as descriptive and hypothesis-generating. Further adequately powered randomized studies are required to clarify its comparative role in clinical practice.

## Supplementary Material

Suppl 1 Pugin’s clinical pulmonary infection score (excluding microbiological criteria).

## Data Availability

The data supporting the findings of this study are available from the corresponding author upon reasonable request.

## Abbreviations

APACHE II: Acute Physiology and Chronic Health Evaluation II
CPIS: Clinical Pulmonary Infection Score
MDR: multidrug-resistant
SOFA: sequential organ failure assessment
TBI: traumatic brain injury
VAP: ventilator-associated pneumonia
WBC: white blood cell count
