# Supplementary material for: Clinical Outcomes of Aerosolized Versus Intravenous Colistin in Ventilator-Associated Pneumonia Caused by Multidrug-Resistant Gram-Negative Bacteria
Source: J Clin Med Res. 2026 Jan 16;18(1):42–9. doi: 10.14740/jocmr6415 (PMC12861513; doi:10.14740/jocmr6415)
Supplement: Suppl 1 — Pugin’s clinical pulmonary infection score (excluding microbiological criteria). [file jocmr-18-01-042-s001.docx]

**Suppl 1.** Pugin’s clinical pulmonary infection score (excluding microbiological criteria)

| **Criteria** | **Score** |
| --- | --- |
| **Body temperature (°C)** |  |
| ≥ 36.5 and ≤ 38.4 | 0 |
| ≥ 38.5 and ≤ 38.9 | 1 |
| ≥ 39 or ≤ 36 | 2 |
| **White blood cell count (cells/mm³)** |  |
| 4,000 ≤ WBC ≤ 11,000 | 0 |
| < 4,000 or > 11,000 | 1 |
| < 4,000 or > 11,000 with band forms ≥ 50% | 2 |
| **Tracheal secretions** |  |
| None or scant | 0 |
| Abundant but not purulent | 1 |
| Abundant and purulent | 2 |
| **Oxygenation (PaO₂/FiO₂, mm Hg)** |  |
| > 240 or presence of ARDS | 0 |
| ≤ 240 without ARDS | 2 |
| **Chest radiograph** |  |
| No infiltrate or no new infiltrate | 0 |
| Diffuse or patchy infiltrate | 1 |
| Localized infiltrate or progression | 2 |
| **Total score** | 0 to 10 |

ARDS: acute respiratory distress syndrome.
